# Effect of Anti-Mullerian Hormone in Culture Medium on Quality of Mouse Oocytes Matured In Vitro

**DOI:** 10.1371/journal.pone.0099393

**Published:** 2014-06-16

**Authors:** Yihui Zhang, Li Shao, Yixin Xu, Yigui Cui, Jiayin Liu, Ri-Cheng Chian

**Affiliations:** 1 State Key Laboratory of Reproductive Medicine, Nanjing Medical University, Nanjing, China; 2 Division of Research, Department of Obstetrics and Gynecology, McGill University, Montreal, Canada; China Agricultural University, China

## Abstract

Anti-mullerian hormone (AMH) is thought to reflect the growth of follicles and the ovarian function. However, the role of AMH in culture medium during in vitro maturation (IVM) on oocyte quality and subsequent development potential is unclear. The objective of this study is to investigate the effect of recombinant human AMH (rh-AMH) supplemented into IVM medium on oocyte quality. Cumulus-oocyte complexes (COCs) were obtained from ICR mice and cultured in vitro with the different concentrations (0–1,000 ng/ml) of rh-AMH. Following 16–18 h of culture, quantitative PCR and ELISA were performed to analyze GDF9 and BMP15 mRNA expression and protein production from the oocytes. Subsequently, in vitro fertilization (IVF) and early embryonic development were employed to further evaluate the quality of in vitro matured oocytes. The results showed that AMH was only expressed in cumulus cells but not in the oocytes. However, AMH most specific receptor, AMHR-II, was expressed in both oocytes and cumulus cells. The levels of GDF9 and BMP15 expression and blastocyst formation rate were significantly increased (p<0.05) when the IVM medium was supplemented with 100 ng/ml of rh-AMH. With AdH1-SiRNA/AMH for knocking down of AMH expression during IVM significantly reduced (p<0.05) the levels of GDF9 and BMP15 expression and blastocysts formation rate. These results suggest that AHM improves oocytes quality by up-regulating GDF9 and BMP15 expressions during IVM.

## Introduction

Anti-mullerian hormone (AMH, also known as Mullerian inhibiting substance [MIS]), belonging to the transforming growth factor β (TGF-β) superfamily [1], is well known for its role in male sexual differentiation [2]. AMH signals through a receptor complex consisting of type I (AMHR-I) and type II (AMHR-II) receptors. AMH and AMHR-II are mutually specific [3], [4]. The formation of the AMH signaling complex is initiated by the binding of AMH to AMHRII, which activates type I receptor (AMHR-I) then phosphorylates the cytoplasmic Sma- and Mad-related proteins (Smads) in concert with other transcription factors to regulate responsive genes [5].

In female, AMH is only secreted by granulosa cells and cumulus cells. As studies have found that AMH functions inhibitory effects on follicle sensitivity to FSH and initial follicle recruitment [4]–[7], AMH has been thought to be an important player in two checkpoints that regulate the efficiency of primordial follicle pool usage and the choice of the dominant follicle: recruitment and selection, respectively. Earlier studies using anti-AMH-deficient mice suggested that AMH is involved in the regulation of primordial follicle recruitment [5]. It also has been demonstrated that AMH inhibits initiation of primordial follicle growth [6]. Therefore, AMH acts as an inhibitory growth factor in the ovary during the early stages of folliculogenesis. Since AMH is mainly produced in the growing ovarian follicles, it has been fully confirmed that serum AMH is positively related to the number of antral follicles, and the value of serum AMH has been widely used in predicting ovarian reverse for infertility treatment [8]–[13].

In addition, some studies found a positive correlation between the levels of AMH in follicular fluid (FF) and rates of fertilization/implantation/clinical pregnancy/high quality embryo in human in vitro fertilization (IVF) and intracytoplasmic sperm injection (ICSI) treatments [14]–[17]. Recently, Mehta et al. [18] reported that there is an inverse correlation of the AMH levels in FF with clinical pregnancy outcomes. However, Anckaert et al. [19] indicated that there is no correlation between the embryo quality and AMH in FF. Therefore, the correlation between the levels of AMH in FF and clinical outcomes is controversial. The role of AMH in affecting oocyte quality is still largely unknown

Immature oocyte retrieval followed by in vitro maturation (IVM) of those immature oocytes is a potentially useful treatment in assisted reproductive technology (ART) and female fertility preservation [20]. However, the clinical pregnancy and live birth rates have been shown lower in IVM oocytes comparing to the conventional gonadotropin stimulated in vivo matured oocytes [21]. It has been also shown that the there is higher early pregnancy losses with IVM oocytes [22]. Although a number of studies attempted to improve oocyte quality by modifying IVM medium, the results met limited success [23]. There is no information available for the effect of AMH supplemented into IVM medium on oocyte quality improvement.

The objective of the present study was to determine the effect of AMH on oocyte quality during IVM evaluated by both molecular evidence and subsequent embryonic development potential following IVF.

## Materials and Methods

### Animals

ICR mice were fed with a standard diet and maintained in a temperature-controlled room (20–22°C), on a 12/12 h light/dark cycle. All experiments were approved by Animal and Human Ethics Board of Nanjing Medical University, and were conducted in accordance with the Animal Research Committee Guidelines of Nanjing Medical University.

### Isolation of Immature Cumulus-Oocyte Complexes (COCs) and *In Vitro* Maturation (IVM)

Immature COCs were obtained from female mice (6–8 weeks of age) stimulated with 10 IU equine chorionic gonadotropin (eCG) (Folligon; Intervet, Castle Hill, Australia). At 44 h post eCG injection, mice were euthanized by cervical dislocation. The ovaries were removed and placed in IVM medium, alpha modification of minimum essential medium (αMEM) (M4526, Sigma, USA) supplemented with 10% fetal bovine serum (FBS) (12003C, Sigma, Australia), 0.25 mM sodium pyruvate (11639, GIBCO, Australia), 50 mIU/ml recombinant human follicle-stimulating hormone (FSH) (F4021, Sigma, USA) and 3 ng/ml epidermal growth factor (EGF) (96-AF-100-15-1000, Peprotech, USA). COCs were isolated by puncturing of ovarian follicles with a needle, collected after washing twice and then subjected immediately to maturation in culture. Ten immature COCs were culture in each 30 µl droplet of IVM medium covered by mineral oil (MKBG7544V, Sigma, USA) at 37°C with 5% CO_2_ in air for 16–18 h [24].

### Immunofluorescence Staining for AMH and AMHR-II

COC samples were fixed in 3–4% paraformaldehyde in phosphate buffered saline (PBS) for 25 minutes at room temperature, and then incubated for 30 minutes with PBS containing 0.01% triton X-100. The fixed and permeabilized COCs were further incubated with 1% bovine serum albumin (BSA)(A2058, Sigma, USA) in PBS for 30 minutes at room temperature, followed by incubating with anti-AMH (1∶50 dilution in PBS, SC- 6886, Santa Cruz, USA), anti-AMHR-II (1∶50 dilution in PBS, SC- 67287, Santa Cruz, USA) antibody in a humidified chamber at 4°C for overnight. COCs were washed with PBS and subsequently incubated with goat anti-rabbit IgG-TRITC (1∶50 dilution, BA1090, Boster, China) or rabbit anti-goat IgG-TRITC (1∶50 dilution, BA1091, Boster, China) for 1 hour at room temperature in dark. As the final step, COCs were incubated with 0.1 g/ml DAPI for 1 minute, and the images were captured with a fluorescence microscope (NIKON TE2000).

### Extraction of RNA from Cumulus Cells and Oocytes/PCR and Real-Time Reverse-Transcription PCR

Total RNA isolation was performed using the RNeasy Micro Kit (145020967, Qiagen, USA) from oocytes and cumulus cells respectively. In-vitro RT-PCR was performed using Sensiscript Reverse Transcription Kit (A2058, Qiagen, USA) and oligo-dT (S0296, Takara, Japan) primer at 37°C for 1 hour. For real-time PCR reaction, cDNA was used as template for amplification to quantify the mRNA concentrations of the tested genes using Quanti Test SYBR Green PCR kits (204145, Takara, Japan). Quantification of gene expression was estimated by the standard curve method and each experiment was repeated at least three times. The PCR products (15 µl) were run on a 1.2% (wt/vol) agarose gel, stained with ethidiumbromide (25 mg/ml). The specific primer sequences were summarized in Table 1.

**Table 1:** Real-time PCR primer sequences.

Gene	GenBank accession no.	Forward primer	Reverse primer	PCR size (bp)
AMH	NM_007445.2	TGCTAGTCCTACATCTGGCTGA	GTCCAGGGTATAGCACTAACAGG	120
AMHRII	NM_144547.2	GCAGCACAAGTATCCCCAAAC	GTCTCGGCATCCTTGCATCTC	204
GDF9	NM_008110.2	TGGAACACTTGCTCAAATCGG	GACATGGCCTCCTTTACCACA	106
BMP15	NM_009757.4	GAAAATGGTGAGGCTGGTAAAG	AGATGAAGTTGATGGCGGTAAA	153
GAPDH	NM_008084.2	GGGTGGTCCAGGGTTTCTTACT	AGGTTGTCTCCTGCGACTTCA	187

### ELISA for GDF9 and BMP15 Proteins

COCs cultured supernatants were collected by centrifugation (20,00 *g* for 10 minutes) and stored at −80°C. The procedure was performed with a GDF9 ELISA Kit (SC-12244, Santa Cruz, USA) and a BMP15 ELISA Kit (E03B0394, Blue Gene, China) following the manufacturer's instructions. OD values were measured at 450 nm using a spectrophotometer (M680, Bio-Rad, USA). The concentrations of GDF9 and BMP15 in the samples were determined by comparing the OD values of the samples to the standard curve.

### Construction of Recombinant Adenovirus

In order to further confirm the role of rh-AMH in regulating oocyte maturation, AdH1-SiRNA/AMH adenoviruses were generated by Sunbio Company (Shanghai, China). For AMH knockdown, recombinant adenoviruses AdH1-SiRNA/AMH expressing siRNA targeting AMH and AdH1-SiRNA/NS (non-silencing) were constructed. The potential target sequences for RNA interference (RNAi) were scanned with the siRNA Target Finder and Design Tool available from the Ambion Website. The selected target sequence (SiRNA/AMH), 5′-CCGGGCAGTTGCTAGTCCTACATTTCAAGAGAATGTAGGACTAGCAACTGCTTTTTTG-3′, corresponded to region 375–393 bp after the AMH start codon. The negative control (SiRNA/NS) sequence was 5′-CCGGTTCTCCGAACGTGTCACGTTTCAAGAGAACGTGACACGTTCGGAGAATTTTTG-3′. These sequences were sub-cloned into adenoviral shuttle vector pShuttle-H1 according to the method used by Shen et al [25]. The pShuttle-H1-siRNA/AMH and pShuttle-H1-siRNA/NS were then recombined with backbone plasmid pAdEasy-1 in BJ5183 bacteria. Adenovirus generation, amplification and titer examinations were performed according to the simplified system described by He et al [26]. Viral titer, determined by plaque assay in 293 cells, was 5.2×10^10^ ifu (infectious units)/ml in AdH1-SiRNA/AMH and 2.4×10^11^ ifu/ml in AdH1-SiRNA/NS.

Verification was performed in mouse granulosa cells before use in this study. After infecting the mouse granulosa cells with AdH1-SiRNA/AMH or the control AdH1-SiRNA/NS adenoviruses for 48 h, more than 80 percent of granulose cells were infected by recombinant adenoviruses andAMH expression was decreased over 80% after infection with AdH1-SiRNA/AMH compared to AdH1-SiRNA/NS (Figure S1). COCs were infected with AdH1-SiRNA with different multiplicity of infection (MOI) (3×10^6^ ifu/COC, 3×10^5^ ifu/COC, 3×10^4^ ifu/COC, 3×10^3^ ifu/COC, 3×10^2^ ifu/COC) for 18 h, and showed that there were more than 90% cumulus infected by recombinant adenoviruses without obvious cell-damaging when cumulus cells were infected by AdH1-SiRNA/AMH with 3×10^4^ ifu/COC. Therefore, COCs were cultured in vitro with AdH1-SiRNA/AMH or AdH1-SiRNA/NS at 3×10^4^ ifu/COC respectively for the further experiments.

### In Vitro Fertilization (IVF) and Embryonic Development Culture

Following 16–18 h of IVM, COCs were washed twice in IVF medium (HTF medium, In Vitro Care, USA), and then 10–15 COCs were placed into 50 µl of droplet IVF medium under mineral oil that were prepared at least 2 h in advance and equilibrated at 37°C in 5% CO_2_ incubator. Epididymal sperm suspensions are prepared from the male ICR mice, and pre-incubated for 60 minutes in IVM medium containing 9.0 mg/ml BSA to ensure sperm capacitation. The final concentration of 2×10^6^ sperm/ml was introduced into 50 µl of droplet contained the maximum of 15 COCs. Sperm and oocytes were incubated together for 6 h, and then the oocytes were washed to place into 20 µl of KSOM+AA medium (MR-121-D, Millipore, USA) droplet under mineral oil for further developmental culture at 37°C a high humidified and 5% CO_2_ incubator. The cleavage rate was confirmed 24 h after insemination, and then the cleaved embryos continued culture until day 5 (120 h post-insemination) without changing the medium. At the end of culture, the percentages of blastocyst formation were assessed, and then provided for differential staining.

### Differential Staining of Blastocysts

Blastocysts were fixed in 4% formaldehyde solution in PBS for 15 minutes at room temperature. They were washed in PBT (with 0.1% Tween 20) and permeabilized with 0.1% Triton in PBS for 15 minutes, followed by treating with primary antibodies overnight at 4°C. Primary antibodies used in this study were OCT4 (1∶200 dilution, 19857, Abcam, USA). Embryos were incubated with goat anti-rabbit IgG-TRITC (1∶50 dilution, BA1090, Boster, China) for 1 h at room temperature in dark. Finally, blastocysts were incubated with 0.1/ml DAPI for 1 min. The nuclei of blastocyst were observed immediately under fluorescence microscope (NIKON TE2000) equipped with a UV filter (with excitation set at 355–530 nm and emission at 465–615 nm with a long pass filter). The nuclei of the ICM were labeled with OCT4 appeared red and the nuclei of the total cell numbers (TCN) were stained with DAPI fluoresced blue.

### Experimental Design

#### Localization of AMH and AMHR II in COCs

AMH works by interacting with specific receptors on the surfaces of the target cells. The best-known and most specific effects of AMH mediate through AMHR II [27]. To understand how AMH influences the quality of oocytes, the expression of AMH and AMHR II in COCs with mRNA levels were examined firstly, and then for the localization of AMH and AMHR-II in COCs with protein level were examined only for the cultured (16–18 hours) COCs by immunofluorescence staining.

#### Effects of different concentrations of rh-AMH on oocyte maturation in vitro

The IVM medium was supplemented with 0, 1, 10, 100 and 1,000 ng/ml of recombinant human (rh)-AMH respectively for IVM. At the end of IVM culture, COCs were denuded with 80 unit/ml hyaluronidase in IVM medium for assessment of oocyte maturity. The oocytes with extrusion of first polar body (1PB) were considered mature at metaphase-II (M-II) stage. Oocyte maturation rates in each group were compared in order to determine the optimal concentration of rh-AMH during IVM. The optimal concentration of rh-AMH during IVM was also further determined by both GDF9 and BMP 15, the key factors in oocyte maturation, in mRNA and protein levels.

#### Changes of GDF9 and BMP15 during IVM with or without rh-AMH

Since both mRNA expression and protein level of GDF9 and BMP15 were significantly higher in IVM medium supplemented with 100 ng/ml of rh-AMH, the changes of time course of GDF9 and BMP15 profiles were further examined during IVM (6, 12 and 18 hours respectively) in order to determine the effect of 100 ng/ml rh-AMH on oocyte quality during IVM.

#### Supplementation of rh-AMH into IVM medium on subsequent embryonic development

To determine the effect of rh-AMH on oocyte quality during IVM, COCs were cultured in IVM medium with or without 100 n/ml of rh-AMH, respectively. Following IVM and IVF, 2-cell stage embryos were recorded on the second day post-insemination, and blastocyst formation rates were observed on day 5 after embryo culture. To further confirm the role of rh-AMH in regulating oocyte quality, COCs were infected with AdH1-SiRNA/AMH or AdH1-SiRNA/NS adenoviruses during IVM. Then the embryonic developments (2-cell and blastocyst stages) in each group were compared following IVF. The quality of blastocysts was evaluated by the ratio of inner cell mass (ICM)/total cell number (TCN) following the differential staining.

### Statistical Analysis

The data were processed for statistical analysis by SPSS 16.0 and are presented as the mean±SD. Statistical comparisons between each group were calculated by independent-sample t-test and p<0.05 were considered statistically significant.

## Results

### Localization of AMH and AMHR II in COCs

**Figure 1:**
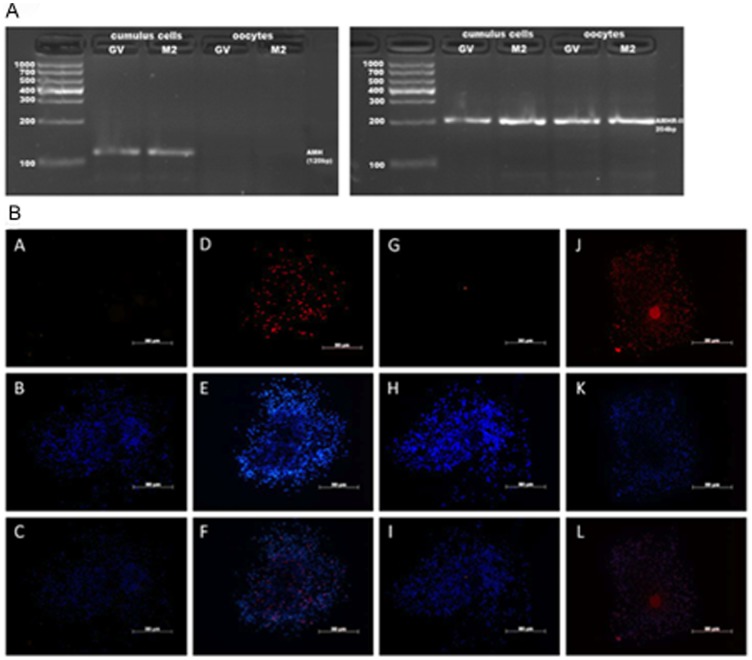
Localization of AMH and AMHR-II by PCR and immunofluorescence staining. (**A**) RNA was isolated from oocytes and cumulus cells from 30 COCs that were at GV stage or at MII stage after IVM, respectively. One-microliter amounts of cDNA were used as templates for PCR. (**B**) COCs after 16–18 h of culture in vitro were stained with mouse isotype IgGs as negative controls of AMH (A) and AMHR-II (G), AMH (D), AMHR-II (J) respectively, followed by TRITC conjugated secondary antibodies and DAPI (B,E,H,K), and merged images (C,F,I,L). Bars = 50 µm.

As shown in Figure 1, AMH was only expressed in cumulus and granulose cells before and after IVM, but not in the oocytes. However, AMHR II was expressed in both oocytes and cumulus cells before and after IVM.

### Effects of Different Concentrations of rh-AMH on Oocyte Maturation In Vitro

**Figure 2:**
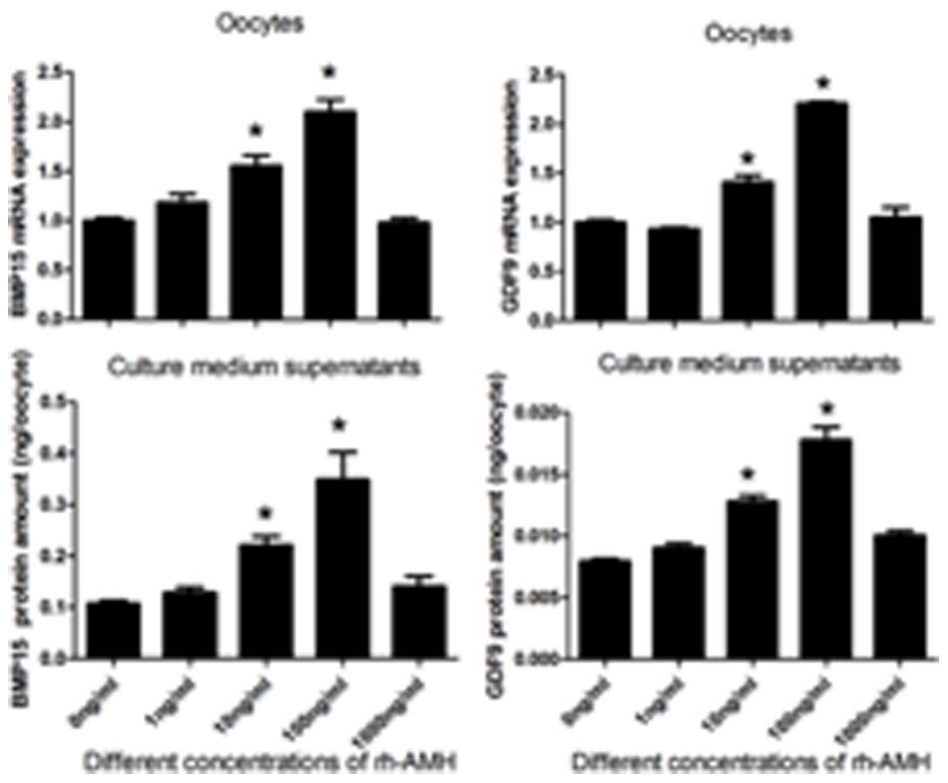
Effect of different concentrations of rh-AMH supplemented into IVM medium on GDF9 and BMP15 mRNA expression and protein production from the oocytes. GDF9 and BMP15 mRNA in the oocytes were measured by RT-PCR, and GDF9 and BMP15 proteins in IVM-medium were measured by ELISA, respectively. Oocytes were harvested at 16–18 h after IVM. *Indicates significant differences (p<0.05) compared to the control group. Data were from 3 replicates.

The oocyte maturation rates were not significantly different among groups when IVM medium was supplemented with 0, 1, 10, 100 and 1,000 ng/ml of rh-AMH for IVM (Table 2). Although there were no differences among groups for oocyte maturation, the expressions of GDF9 and BMP15 in oocytes and the levels of protein in IVM medium were a dose-dependent manner when rh-AMH at concentrations of 1–100 ng/ml. However, the expressions of GDF9 and BMP15 in the oocytes and the protein levels in IVM medium were significantly down-regulated at concentration of 1,000 ng/ml of rh-AMH during IVM compared to 100 ng/ml of rh-AMH group (Figure 2).

**Table 2:** The effect of different concentrations of rh-AMH in IVM-medium on mouse oocyte maturation following IVM.

Concentration of rh-AMH (ng/ml)	No. of COCs examined	No. of oocytes matured (%, mean±SD)*
0	115	111 (96.6±4.1)
1	114	108 (94.8±4.5)
10	112	105 (93.8±1.9)
100	111	107 (96.1±6.1)
1,000	118	110 (93.2±2.6)

Data showed with 6 replicates.

*There are no significant differences among groups.

### Changes of GDF9 and BMP15 during IVM with or without rh-AMH

**Figure 3:**
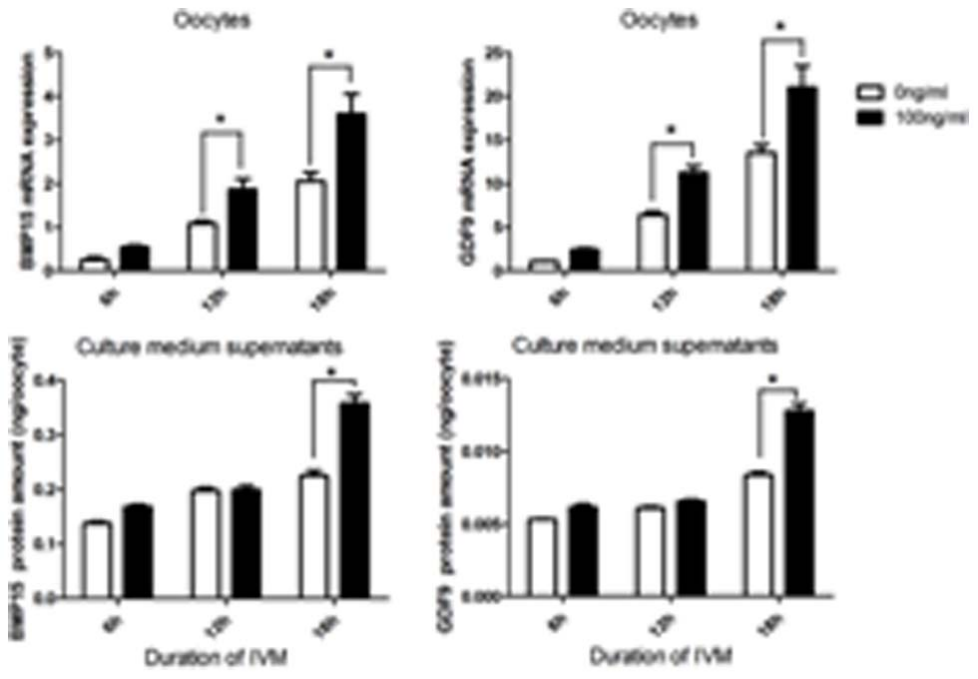
Effect of IVM medium supplemented with or without 100/ml rh-AMH on GDF9 and BMP15 mRNA expression and protein production from the oocytes following IVM at different time points. GDF9 and BMP15 mRNA expression in the oocytes were measured by RT-PCR, and GDF9, and BMP15 proteins in IVM-medium were measured by ELISA, respectively. Oocytes and IVM-medium supernatants were collected at 6, 12 and 18 h respectively during IVM. *Indicates significant differences (p<0.05) compared to the control group. Data were from 3 replicates.

As shown in Figure 3, although there were no differences in both gene expressions and protein levels of GDF9 and BMP15 in the oocytes and IVM medium until COCs cultured to 6 h, the expressions of GDF9 and BMP15 were significantly increased (p<0.05) following 12 h of culture with rh-AMH compared to without rh-AMH in IVM medium. The levels of protein of GDF9 and BMP15 in IVM medium were significantly higher (p<0.05) when COCs were cultured with rh-AMH than without rh-AMH following culture at 18 h point.

### Supplementation of rh-AMH into IVM Medium on Subsequent Embryonic Development

**Figure 4:**
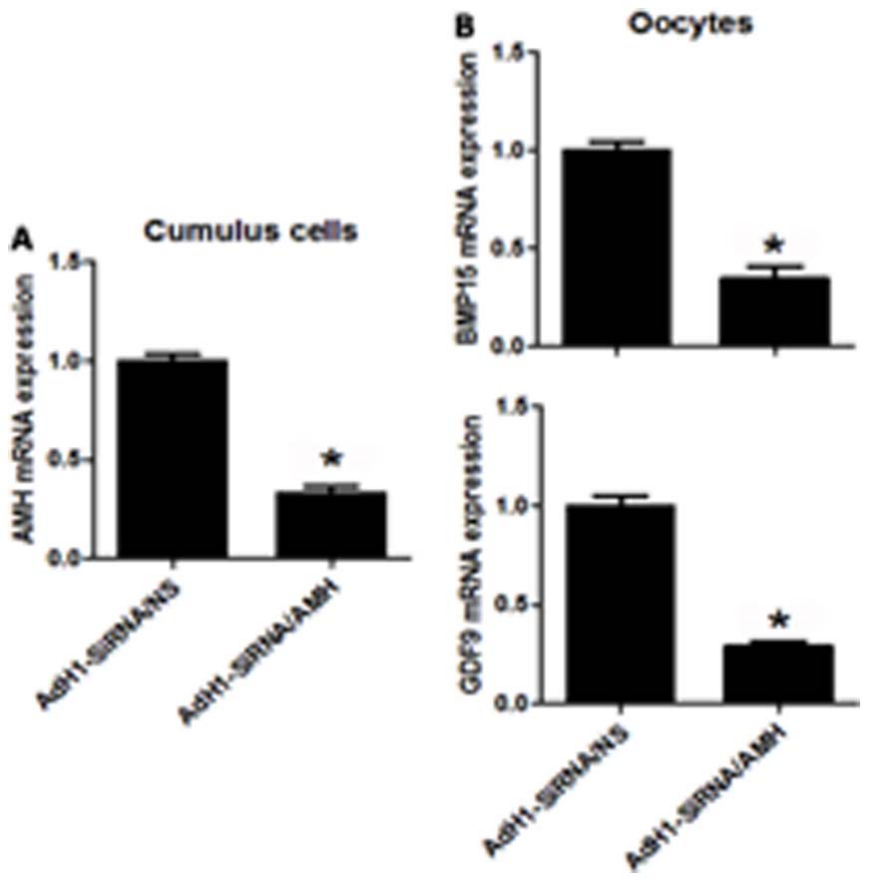
Effect of AMH knockdown on GDF9 and BMP15 mRNA expression from mouse cumulus cells and oocytes. Cumulus cells and oocytes were harvested from COCs infected by AdH1-SiRNA/NS or AdH1-SiRNA/AMH during IVM, respectively. (A) Shows AMH mRNA expression in cumulus cells infected by AdH1-SiRNA/NS or AdH1-SiRNA/AMH; (B) Shows GDF9 and BMP15 mRNA expressions in oocytes from COCs infected by AdH1-SiRNA/NS or AdH1-SiRNA/AMH, respectively. *Indicates significant differences (p<0.05) compared to the control group. Data were from 3 replicates.

As shown in Figure 4A, the expression of AMH mRNA was significantly reduced (p<0.05) by AdH1-SiRNA/AMH adenovirus in cumulus cells. With AMH knocking down by AdH1-SiRNA/AMH, both BMP15 and GDF9 mRNA expressions were down-regulated significantly (p<0.05) (Figure 4B).

Table 3 shown, although there were no significant differences in the cleavage rates among groups, the blastocyst formation rate was significantly higher (p<0.05) in IVM medium supplemented with 100 ng/ml of rh-AMH group compared to the control group. Evidently the blastocyst formation rate was significantly decreased (p<0.05) in IVM medium treated AdH1-SiRNA/AMH group compared to AdH1-SiRNA/NS group.

**Table 3:** Subsequent embryonic development of immature COCs matured in IVM-medium without rh-AMH (Control) or with 100 ng/ml rh-AMH, AdH1-SiRNA/NS, AdH1-SiRNA/AMH respectively and followed by in vitro fertilization (IVF).

Group of treatment	No. of COCs examined	No. of 2-cell embryos developed (%)	No. of blastocysts developed (%)
Control	157	120 (76.4±5.4)	38 (31.7±2.7)
rh-AMH	150	120 (80.0±4.5)	48 (40.0±3.3)*
AdH1-SiRNA/NS	142	103 (73.1±8.9)	35 (33.9±5.9)
AdH1-SiRNA/AMH	135	95 (70.3±5.9)	20 (20.6±2.9)**

Data showed 3 replicates.

*Indicates significant difference compared to control group (P<0.05);

**indicates significant difference compared to AdH1-SiRNA/NS group (P<0.05).

**Figure 5:**
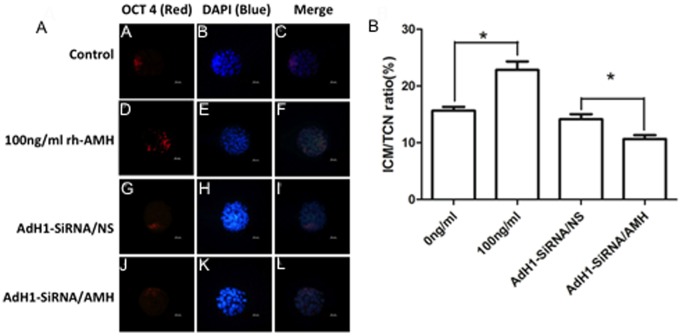
Blastocyst quality was evaluated by the ratio of ICM/TCN. (**A**) The blastocysts derived from the oocytes matured in vitro without rh-AMH (A–C) or with rh-AMH (D–F), and treated with AdH1-SiRNA/NS (G–I) and AdH1-SiRNA/AMH (J–K) respectively were stained with OCT4 for ICM (A, D, G, J), DAPI for TCN (B, E, H, K) and merged images (C, F, I, L). Bars = 20 µm. (**B**) The ratio of ICM/TCN represents by mean±SD in each group with 6 replicates. * Indicates significant differences (p<0.05) between the groups.

The quality of embryos was evaluated by ICM/TCN (Figure 5A). The ratio of ICM/TCN was significantly higher (p<0.05) in IVM medium with rh-AMH compared to without rh-AMH (Figure 5B). This was further confirmed by knocking down AMH with AdH1-SiRNA/AMH treated IVM medium group which significantly reduced (p<0.05) the ratio of ICM/TCN compared to AdH1-SiRNA/NS treated IVM medium group (Figure 5B).

## Discussion

The results of the present study demonstrated that AMH is only expressed in cumulus and granulose cells, but not in the oocytes and that AMHR II is expressed in both oocytes and cumulus cells. The results of the present study also demonstrated that the addition of 100 ng/ml of rh-AMH to IVM medium improves oocyte quality and that oocyte GDF9 and BMP15 expressions are down-regulated when COCs were treated by AdH1-SiRNA/AMH during IVM, resulting in reduced blastocyst formation rate. Therefore, these results suggest that AMH improves oocytes quality by up-regulating GDF9 and BMP15 mRNA expressions during IVM.

Studies have shown that AMH expression is strongest in pre-antral and small antral follicles, and then decreases continually [28]. AMHR-II is co-expressed with AMH in granulosa cells of growing follicles [29], [30]. In rat and sheep, AMH and AMHR-II are specifically expressed in granulosa cells, and there is no expression in the oocytes, theca and interstitial cells or expressed very little amount of AMH and AMHR-II in those cells [30]–[35]. Interestingly, our results indicated that AMH mRNA expression and protein only localized in cumulus cells (Figure 1). However, AMHR-II mRNA expression and protein were localized at both oocyte and cumulus cells.

AMH may have effects on both cumulus cells and oocytes through autocrine and paracrine when COCs were cultured in vitro. In rat [34] and sheep [35], the expression of AMHR-II was not observed in the oocytes from antral follicles. Therefore, the different observations could be due to technical variations between the studies or due to different species. In addition, as AMHR-II is a membrane receptor, using immunocytochemical method may not detect AMHR-II expression, because the cell membranes, especially large cells like oocytes, it may not be integrated properly in these studies. It has been reported that another member of TGF-β superfamily, BMP receptor IB (BMPR-IB) is located at both oocyte and granulosa cells in sheep ovary [36]. Same as sheep ovary, AMHR-II may be similar to this TGF-β superfamily receptor in mouse COCs. However, Sedes L et al. [37] found that AMH could recruit BMPR-IA in immature granulosa cells. Therefore, further study is required to confirm the linkage of those two receptors.

Belonging to the TGF-β superfamily, GDF9 and BMP15 play crucial roles in the follicular development, ovulation, oocyte maturation, and embryo development [38]–[41], and they are essential factors for folliculogenesis and female fertility [42]. It has been reported that GDF9 and BMP15 are closely associated with oocyte quality and embryo developmental potential [43]. In GDF9-deficient female mice, the ultrastructure of oocytes is abnormal; ovulation and oocyte fertilization rate are decreased in BMP15 knock-out model [44]. Also it has been reported that addition of exogenous GDF9 and BMP15 to IVM medium during IVM could increase blastocyst yield following IVF [45]. Since GDF9 and BMP15 are key factors that regulate oocyte maturation for subsequent embryo developmental potential, we have employed those two factors as molecule marker to evaluate oocyte quality in our study.

The concentration of AMH in mouse follicular fluid is still unclear, because mouse follicles are too small to aspirate its fluid. Therefore, we chose the range of AMH concentrations of human follicular fluid for our experiments. The concentrations of AMH in human small antral follicles (mean 790 ng/ml) were almost three orders of magnitude higher than in pre-ovulatory follicles (mean 1.17 ng/ml) [46]. AMH levels in follicular fluid, selected from follicles 4–8 mm in diameter, were significantly higher in women with anovulatory polycystic ovary syndrome (PCOS) (median 466.2 ng/ml) compared with normal-ovulatory controls (median 78.0 ng/ml) [47]. In addition, we chose these concentrations according to some other studies adding AMH into culture medium when cultured follicles or ovaries in vitro [48]–[50].

In rodents, AMH plays a decelerating role in the process of primordial follicle recruitment and follicular maturation [13]. Concerning possible effects of AMH on oocyte maturation, conflicting data have been reported. One study has shown that AMH inhibits oocyte meiosis in rat [51], but another study indicated that there is no effect of AMH on oocyte meiosis [52]. The results of our study confirmed that AMH has no inhibitive effect on oocyte meiosis with concentrations of 0–1,000 ng/ml (Table 2). In contrast, the concentration of 100 ng/ml of rh-AMH in IVM medium improves the oocyte quality in terms of GDF9 and BMP15 mRNA expression and protein secretion (Figure 2) as well as blastocyst formation rate and blastocyst quality (Table 3 and Figure 5). Interestingly, we found that levels of these key transcripts in the oocytes up-regulated with AMH following time course pattern during IVM (Figure 3), and down-regulated by AdH1-SiRNA/AMH when cumulus cells were infected by adenovirus during IVM (Figure 4). This result indicated that supplementation of rh-AMH into IVM medium improves oocyte quality during IVM.

In reproductive medicine, a serum AMH level has been recognized to be a useful diagnostic and prognostic tool. Since serum AMH is positively correlated to the number of antral follicles, the levels of serum AMH have been used as a reliable marker for the ovarian reserve [8] and a predictor of the ovarian response to controlled ovarian hyper-stimulation [53]. However, it still is unclear how AMH and FSH correlated during follicular development and oocyte maturation [28], [54], [55]. Currently, the mechanism behind this is largely unclear, so it needs further study to find out how AMH exerts its function associated with FSH and growth factors, such as EGF, during these processes.

In conclusion, the results of this study indicate that supplementation of 100 ng/ml of rh-AMH into IVM medium together with FSH and EGF improves oocyte quality. These results also indicate that rh-AMH improves oocytes quality by up-regulating GDF9 and BMP15 expression during IVM. These results suggest that IVM medium may be required rh-AMH supplementation during IVM in order to increase oocyte development competence.

## Supporting Information

Figure S1 **AMH knockdown experiments with recombinant adenoviruses AdH1-SiRNA/AMH targeting to AMH and with recombinant adenoviruses AdH-SiRNA/NS as control.** Since the recombinant adenoviruses contain a green fluorescent protein (GFP) gene, GFP expression was visualized by fluorescence microscopy after transfected to granulosa cells with AdH1-SiRNA/AMH and AdH1-SiRNA/NS for 48 h. As shown in Figure S1A, more than 80 percent of granulose cells expressed green fluorescent light, which means these cells were infected by recombinant adenoviruses. These cells also were used for Western blot and the results were shown in Figure S1B. GAPDH was used as the standardized reference. The expressions of AMH were different in the granulosa cells which were infected by AdH1-SiRNA/AMH and AdH1-SiRNA/NS respectively. As shown in Figure S1 C, the expression of AMH was decreased over 80% after infection with AdH1-SiRNA/AMH for 48 h compared to AdH1-SiRNA/NS. The experiments were repeated three times. *Indicates significant differences (p<0.05).
(TIFF)
