# Supplementary figures and images for: Effect of Anti-Mullerian Hormone in Culture Medium on Quality of Mouse Oocytes Matured In Vitro
Source: PLoS One. 2014 Jun 16;9(6):e99393. doi: 10.1371/journal.pone.0099393 (PMC4059625; doi:10.1371/journal.pone.0099393)

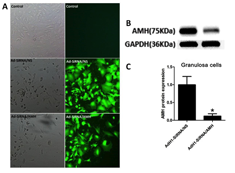

Supplement: Figure S1 — AMH knockdown experiments with recombinant adenoviruses AdH1-SiRNA/AMH targeting to AMH and with recombinant adenoviruses AdH-SiRNA/NS as control. Since the recombinant adenoviruses contain a green fluorescent protein (GFP) gene, GFP expression was visualized by fluorescence microscopy after transfected to granulosa cells with AdH1-SiRNA/AMH and AdH1-SiRNA/NS for 48 h. As shown in Figure S1A, more than 80 percent of granulose cells expressed green fluorescent light, which means these cells were infected by recombinant adenoviruses. These cells also were used for Western blot and the results were shown in Figure S1B. GAPDH was used as the standardized reference. The expressions of AMH were different in the granulosa cells which were infected by AdH1-SiRNA/AMH and AdH1-SiRNA/NS respectively. As shown in Figure S1 C, the expression of AMH was decreased over 80% after infection with AdH1-SiRNA/AMH for 48 h compared to AdH1-SiRNA/NS. The experiments were repeated three times. *Indicates significant differences (p<0.05). (TIFF) [file pone.0099393.s001.tiff]
